# Modulation of social behavior by distinct vasopressin sources

**DOI:** 10.3389/fendo.2023.1127792

**Published:** 2023-02-13

**Authors:** Nicole Rigney, Geert J. de Vries, Aras Petrulis

**Affiliations:** Neuroscience Institute, Georgia State University, Atlanta, GA, United States

**Keywords:** vasopressin, social behavior, bed nuclei of the stria terminalis, sex differences, hypothalamas, social communication, anxiety, neuropeptide

## Abstract

The neuropeptide arginine-vasopressin (AVP) is well known for its peripheral effects on blood pressure and antidiuresis. However, AVP also modulates various social and anxiety-related behaviors by its actions in the brain, often sex-specifically, with effects typically being stronger in males than in females. AVP in the nervous system originates from several distinct sources which are, in turn, regulated by different inputs and regulatory factors. Based on both direct and indirect evidence, we can begin to define the specific role of AVP cell populations in social behavior, such as, social recognition, affiliation, pair bonding, parental behavior, mate competition, aggression, and social stress. Sex differences in function may be apparent in both sexually-dimorphic structures as well as ones without prominent structural differences within the hypothalamus. The understanding of how AVP systems are organized and function may ultimately lead to better therapeutic interventions for psychiatric disorders characterized by social deficits.

## Introduction

The neuropeptide arginine-vasopressin (AVP) has many peripheral functions, such as maintenance of blood pressure and antidiuresis (1, 2). In addition to these well-established physiological effects, AVP (and its non-mammalian analogue vasotocin) has long been implicated in the regulation of social behavior and communication in vertebrates, including humans (3–8). AVP acts on various brain regions to regulate social recognition (9–11), communication (3, 12, 13), aggression (14), maternal care (15), pair bonding (16), and cognition (17). Additionally, AVP contributes to avoidance and anxiety-like behavior in response to stressful situations (18, 19). In humans, AVP has been implicated in psychopathology, as variations in the vasopressin V1a receptor (V1aR) gene and AVP serum levels are associated with autism spectrum disorder (ASD) (20, 21). As such, there has been increasing interest in AVP mechanisms that regulate social behavior, thus opening up translational opportunities (8).

AVP-like-peptides contain conserved features that evolved over half a billion years ago and can be traced to similar molecules in invertebrates (22, 23). In non-mammalian vertebrates, vasotocin (AVT) is the most common ancestral AVP-like molecule and is produced in birds, fish, amphibians, and reptiles, while AVP is produced in most mammalian species. Other variants of AVP, such as lysine vasopressin and phenypressin, are found in marsupials and a limited number of eutherian mammals (24–26). In this review, we will use the term “AVP” for all these peptides. In mammals, AVP acts on three canonical receptor types: V1aR, V1bR, and V2R (27), as well as on the oxytocin receptor (OXTR) (28, 29), with V1aR and OXTR being the predominant receptors expressed in the nervous system. AVP acts on three canonical receptor types: V1aR, V1bR, and V2R (27), as well as on the oxytocin receptor (OXTR) (28, 29), with V1aR and OXTR being the predominant receptors expressed in the nervous system. Furthermore, AVP can have dual actions on cells in target zones *via* V1aR action. For example, AVP causes both direct excitation and indirect inhibition within the lateral septum (LS), in the latter case *via* excitation of inhibitory interneurons (30). In mammals, AVP is produced primarily in the paraventricular nucleus of the hypothalamus (PVN), supraoptic nucleus (SON), and the suprachiasmatic nucleus (SCN). AVP expressed in these nuclei regulate homeostatic functions, such as water/salt balance, blood pressure (i.e., PVN/SON), and circadian rhythms (i.e., SCN). Additional AVP-producing cells are found in the anterior hypothalamus (AH), nucleus circularis (NC), preoptic area (POA), as well as within sensory systems, such as the olfactory bulb (OB) and retina (31). Most mammals also produce AVP within the extended amygdala (bed nucleus of the stria terminalis (BNST), medial amygdala (MeA)), and do so in a steroid-dependent and sex-specific manner (32). Other non-mammalian species (e.g., roughskin newt) have AVP-expressing cells in other brain regions (33), and fish may lack the BNST/MeA AVP system altogether [but see (34)].

While the role of brain AVP on social and other behaviors has been well-described, the linkage of specific AVP sources to specific effects of AVP on social behavior has been, until recently, been based predominantly on circumstantial and correlational evidence, often with varying results sometimes in contradiction with each other. For example, AVP immunoreactivity, measured by immunohistochemical detection of AVP fiber density, and V1aR expression in the septum, detected by autoradiography, is positively correlated with aggression levels in California mice (Peromyscus) (35), but the density of septal AVP-immunoreactive (AVP-ir) fibers is negatively correlated with male aggression in laboratory mice (M. musculus) and rats (36, 37). Additionally, more aggressive rats release less (38) or more (39) AVP in the septum, and bouts of aggression in deer mice (Peromyscus) are positively correlated with BNST AVP-ir cell number (40). In addition, BNST AVP neurons of male laboratory mice increase Fos expression in response to sexual-, but not aggression-related stimuli (41). Direct comparisons of correlational measures and RNA-interference manipulations in birds have yielded different interpretations as to how AVP influences avian social behavior (42). These differences in the relationships between AVP expression, release, cell activity, and aggression, while possibly due to species and other differences, make it clear that studies that investigate the causal nature of AVP action are needed, such as those that delineate which AVP cell populations contribute to AVP effects on social behavior.

**Figure 1:**
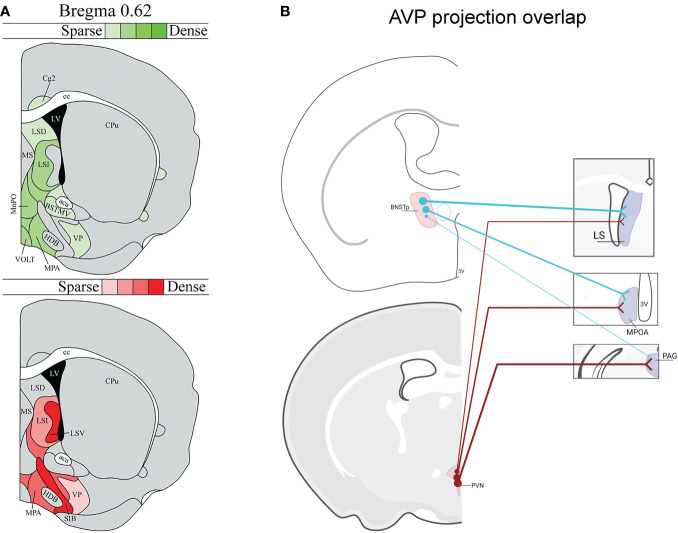
*Vasopressin (AVP) projection site overlap in mice*. **(A)** Image modified from (46) demonstrating that the LS and ventral forebrain receives steroid-dependent AVP signal likely from BNST/MeA as well as from the hypothalamus. Red shading indicates location of AVP-ir fibers that decrease after gonadectomy in males. Green shading indicates location of AVP-ir fibers likely originating from PVN, SON, or accessory cells (i.e., AVP-ir remained after gonadectomy and SCN lesions). AVP-ir fiber density is indicated by color shade. **(B)** Examples of several regions from (46) that receive both BNST and PVN AVP input: lateral septum (LS), medial preoptic area (MPOA), periaqueductal gray (PAG) (not all overlap regions are listed). Thickness of lines represents the strength of AVP fiber projections. Created with BioRender.com.

As most of our foundational knowledge of AVP’s influence on social behavior has been gained from pharmacological targeting of AVP receptors within different brain regions as well as from microdialysis measurements of local AVP release during social behavior (12, 15, 43–45), we have incomplete understanding about which AVP cell populations are directly regulating social behavior. Indeed, the diverse anatomy of AVP projections across species suggests that AVP control of social behavior is complex (42). An additional complication is that distinct AVP sources overlap in their projections to some terminal zones. For example, the LS receives AVP input from both the BNST and PVN (46, 47) (**Figure 1**), and so pharmacological manipulations within the LS may influence multiple AVP pathways, each with potentially different functions. This may be one reason for inconsistent results of manipulations of V1aR in the LS on intermale aggression and anxiety (38, 48) (**Figure 1**). Similarly, microdialysis measurements of AVP release in LS similarly cannot distinguish between AVP derived from different sources (38, 39, 49, 50).

In addition to the adjacent or overlapping innervation of target structures from different AVP sources, complications arise from primarily focusing on targets of AVP action and not on the sources of AVP. For example, somatodendritic release of AVP from magnocellular PVN and SON neurons may generate both local autocrine/paracrine effects and as well as influencing distal regions (51), but see (52). Thus, AVP action on a particular brain region can originate from multiple sources, both synaptic and extrasynaptic. Moreover, the known cross-talk between AVP and OXT, a closely related peptide (29, 53), on their cognate receptors suggests that AVP may have some of its behavioral action *via* OXTR, further complicating the analysis of sources and sites of AVP action (12, 23, 45, 54). Consequently, this review will assess the role of specific AVP cell groups in social behavior by focusing on experiments that directly manipulate these cell populations. Specifically, we will examine the functional role of sexually dimorphic AVP cells within the BNST and MeA, AVP cells within the hypothalamus, and AVP cells in sensory systems, such as the retina and olfactory bulb. For more in-depth information on AVP action within specific target brain regions, see: (3, 12, 22, 44, 49).

## Sexually differentiated vasopressin expression within the bed nucleus of the stria terminalis and medial amygdala

**Figure 2:**
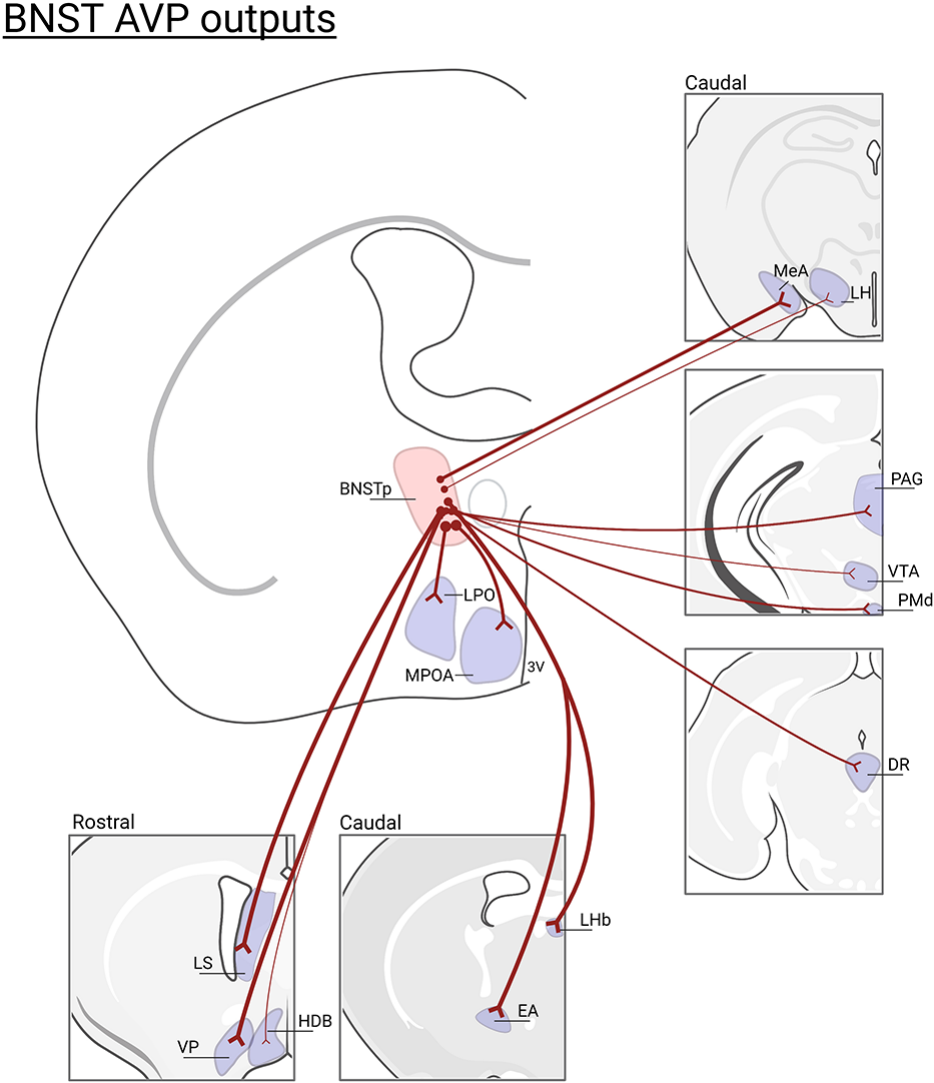
*BNST AVP cell outputs*. Brain regions that lose AVP innervation following gonadectomy (46) or BNST lesions (47, 64): (LS), lateral septum; (VP), ventral pallidum; (HBD)horizontal diagonal band; (MPOA) medial preoptic area; (LPO) lateral preoptic area; (LHb) lateral habenula; (EA) extended amygdala; (MeA) medial amygdala; (LH) lateral hypothalamus, (PAG) periaqueductal gray; (VTA) ventral tegmental area; (PMd) dorsal premammillary nucleus; (DR)dorsal raphe. Not all BNST AVP cell output regions are listed. Thickness of lines represents the density of AVP-ir fiber innervation. Created with BioRender.com.

The BNST and MeA, which is where the sexually-dimorphic and steroid-sensitive AVP cell populations are found, belong to the “extended amygdala” (55, 56) and are key nodes connecting the Social Behavioral Neural Network (SBNN) and the Social Decision-Making Network (57, 58). Work by de Vries and others demonstrated that these cells are greater in number and express more AVP per cell in males than in females (59, 60). They are also steroid-dependent and express only AVP in the presence of gonadal hormones (61), which may act directly on these cells, as they express both androgen receptor (AR) and estrogen receptor alpha (ERα) (62, 63). AVP-expressing cells in the posterior BNST/MeA are the most likely sources of sexually-dimorphic and steroid-dependent AVP innervation of specific brain regions that regulate various aspects of social behavior (e.g., the lateral septum, ventral pallidum, dorsal raphe, lateral habenula, and other areas; see **Figure 2**) (32). Although the BNST and MeA are complex structures with numerous subdivisions (65, 66), AVP cell populations are generally limited to the intermediate and principal posterior sections of BNST and the posterodorsal part of MeA (46, 47). Steroidal effects on AVP peptide and AVP mRNA expression occur during early development as well as in adulthood, reflecting organizational and activational effects of steroid hormones, respectively (67, 68). Additionally, phenotypically female mice with XY sex chromosomes (compared to XX) have denser AVP-ir fibers in the LS compared to XX female mice, demonstrating that genes on sex chromosomes, independent of gonadal development, partially regulate the sexually differentiation of BNST/MeA AVP expression (69).

## Function of BNST AVP cells

### Opposite-sex interactions (affiliative behavior)

The BNST/MeA AVP cells have been linked to control of affiliative or prosocial behavior, primarily in males. For example, when male zebra finches and prairie voles pair-bond with females, Avp mRNA increases in the BNST (68, 70), and male finches that fail to court females have fewer BNST AVP-ir cells (71). Based on these findings and on the observation that BNST AVP cells show Fos expression in the presence of females, but not other rewarding stimuli, Goodson and colleagues argued that BNST AVP is important for positively-valenced social interactions in birds (71). This idea was further supported by BNST AVP cells in roosters and male mice expressing more Fos after copulation, but not, or less so, following aggressive interactions (41, 72). Similarly, BNST AVP neurons of male brown anole lizards show Fos activation during sexual behavior, while PVN AVP neurons show more Fos activation during aggressive encounters (73).

The monogamous prairie vole expresses higher levels of V1aR in the VP and LS compared to other, non-monogamous, vole species, suggesting that this difference in receptor density may contribute to the differences in social behavior between vole species (16, 74, 75). Indeed, blockade or knockdown of V1aR in these brain regions prevents pair bonding and bi-parental behavior in male prairie voles (76–78) and overexpression of Avpr1a in the VP of both prairie and meadow voles facilitates pair bonding behavior (79, 80). Although rats do not form monogamous pair bonds, inserting the prairie vole Avpr1a transgene in LS cells increased social interactions (81). Overall, it appears that AVP action within the LS and VP facilitates pair bonding and social interactions. However, the AVP sources regulating these behaviors have not been conclusively demonstrated.

### Same-sex social interactions (mate competition and aggression)

In addition to its effects on prosocial behaviors, AVP can influence male aggression, but in a way that depends on social context and often differs between species. For example, AVP release in the lateral habenula, a major BNST target, may regulate territorial scent marking in mice (13, 82) and AVP release in the lateral septum (LS), another major BNST target, facilitates aggression in male mice (83) as well as in gregarious zebra finches that compete for a mate, whereas LS AVP infusions inhibit resident-intruder aggression in territorial bird species (42). However, blocking V1aR reduces competition for mates in territorial bird species, while not affecting territorial aggression (84). Indeed, AVP cell groups in the BNST may be responding to female stimuli in the context of mate competition, but not to other social stimuli in the context of territorial aggression or social aversion (85). The activity patterns in BNST AVP cells may also depend on social organization, as BNST AVP-Fos colocalization increases after male-male interactions in gregarious bird species, but decreases after such interactions in more territorial/asocial bird species (85). This pattern suggests that increased BNST AVP cell activity in more social birds may reflect positively-valenced male-male interactions compared to typically more negatively-valenced interactions in more territorial species (85). In addition, the number of BNST AVP cells positively correlate with aggression in California mice (35, 40, 86). In this species, increased paternal care can increase both territorial aggression and the number of BNST AVP-ir cells (87). BNST AVP may also influence female aggressive behavior as AVP injections within the LS reduces female resident-intruder aggression (88).

### Social memory

AVP can also influence social memory through its actions on the putative targets of BNST/MeA AVP cells, such as the LS (89). In rats, V1aR antagonist injected into the LS impaired social memory in males but failed to do so in females when injected peripherally or intracerebroventricularly (9, 90, 91). V1aR antagonist injected directly into the LS, however, can block social recognition in adult males and female rats (11), but only in males while juvenile (11). These effects of V1aR antagonist are modulated by gonadal steroids in both rats and mice (9, 10). This sex- and steroid-dependency of AVP action suggests that projections from BNST (and/or MeA) cells are the most relevant sources of AVP acting on social recognition (9, 10, 90, 92). Indeed, removal of BNST AVP cells in mice show that these cells are necessary for social recognition in males, but not females (see below) (93).

### Developmental aspects of social behavior (social play)

Social play fighting in juvenile rats, an important behavior for social skill development, is mediated by V1aR action within the LS in a sex-specific manner. Specifically, pharmacological blockade of V1aR in the LS increased social play behavior in juvenile male rats but reduced social play behavior in females (94, 95), and a similar effect was found in the ventral pallidum (96). Therefore, in rats, AVP action within the LS and ventral pallidum may prevent social play in juvenile males, while facilitating social play in juvenile females. Additionally, Avp mRNA expression in the BNST correlates negatively with social play behavior in male, but not female, juvenile rats (97). Although the BNST and MeA (as well as PVN) send AVP projections to the LS and VP (32, 98), future studies are needed to dissect precisely which AVP sources regulate social play.

### Anxiety and social interactions

Since AVP has been implicated in the physiological stress response (99), studies have examined AVP’s role in generating anxiety-like behavior, which can greatly impact how animals interact socially (100). Indeed, V1aR knockdown within the LS reduced anxiety-like behavior in rats (101) and anxiety-like behavior is reduced only in V1aR KO male, but not female, mice (102, 103). These results suggest that the sexually dimorphic BNST/MeA AVP system regulates anxiety-like states primarily in males (104). Since AVP plays a larger role in the regulation of male anxiety-like behavior, this system may influence how males interact with conspecifics, coping strategies, and social memory formation (105).

## Direct manipulation of BNST AVP cells

Despite the substantial indirect evidence implicating the sexually-dimorphic BNST AVP cells in the control of male-typical social behavior, direct evidence for their involvement has been limited. To address this issue, we used intersectional genetic techniques to selectively delete BNST AVP cells in mice and found that such lesions strongly reduced male-male social investigation, modulated aspects of male social communication (i.e., urine marking), and impaired female sexual behavior, without altering resident-intruder aggression, ultrasonic vocalizations, male copulation, or anxiety-related behaviors (106). Similarly, shRNA knockdown of AVP within the BNST also reduced male-male social investigation but, unlike BNST AVP cell ablations, decreased consummatory aspects of male copulatory behavior (107). Some of these effects align with the effects of similar manipulations in birds (42). For example, in territorial Angolan blue Waxbills, knockdown of BNST AVP reduces social contact primarily between males but does not affect anxiety-like behavior (108). However, in the more social zebra finch, knockdown of BNST AVP reduces preference for larger flocking groups (gregariousness), and increases anxiety-like behavior (109). In housing situations that promote mate-guarding, BNST AVP knockdown in zebra finches increases aggression and reduces courtship communication, primarily in males (7, 109). Thus, although species/context differences are apparent, reducing BNST AVP seems to primarily influence male social interactions, mostly (but not exclusively) in the context of male-male competition, communication, and courtship behavior. This suggests that BNST AVP neurons normally play a prominent role in driving specific aspects of male-male competitive behavior and investigation as well as affiliation (i.e., copulatory behavior; gregariousness in birds). In addition, BNST AVP cells are also critical for male social recognition, as deletion of these cells reduces social recognition in males, but not females (93). This is consistent with AVP effects within LS on social recognition (see above) and the recent finding that stimulation of BNST AVP terminals in LS restores social recognition in male mutant mice (Magel2-KO) with pre-existing social recognition deficits (110).

## Function of MeA AVP cells

The MeA is a heterogeneous structure that is implicated in the control of defensive responses, aggression, parental behavior, play behavior, social communication, sexual behavior, as well as social recognition (65, 111). The posterior dorsal part of the MeA (MeApd) contains the steroid-sensitive and sexually dimorphic AVP cell population (32, 47). The MeA and BNST may form an integrated system originating from a singular structure as there is developmental continuity between these two cell groups around the internal capsule (55, 112) and AVP expression is similarly modulated by sex-steroids in these two structures (46). Despite having been described several decades ago (113, 114), few studies have tested the functional role of this AVP cell population. Most of the existing work has focused on the role of MeA AVP cells in regulating defensive responses to stressors, such as predator odors, as well as reproductive behavior. For example, MeApd AVP cell activation (Fos expression) in rats positively correlates with male investigation of female conspecifics and copulatory behavior (115) and reduced methylation of the Avp promoter in MeA/BNST, which promotes gene transcription, correlates with reduced aversion to predator odors (116). Similarly, ablation of MeA AVP cells increases aversion to predator odors whereas overexpression of AVP in MeA reduces aversion to predator odors (117). Counter-intuitively, over-expression of AVP in MeA increases activation of PVN-AVP neurons following predator odor exposure, suggesting that MeA AVP may facilitate PVN AVP cell responses during an acute stressor (118). However, while the functional nature of this interaction is unknown, deletion of PVN AVP cells increases anxiety-like behavior in male mice ((119); see below), suggesting that activation of PVN AVP cells may be normally anxiolytic, and therefore anti-defensive, in males. Taken together, this data suggests that MeA AVP cells normally suppress defensive behavior, perhaps in situations where defensive behavior would be counter-productive, such as in reproductive contexts or during competitive behavior (120). More experiments targeting these cells are clearly needed to determine the social role of MeA AVP cells, their projections, and neurochemical interactions.

## Vasopressin cells of the paraventricular nucleus of the hypothalamus

**Figure 3:**
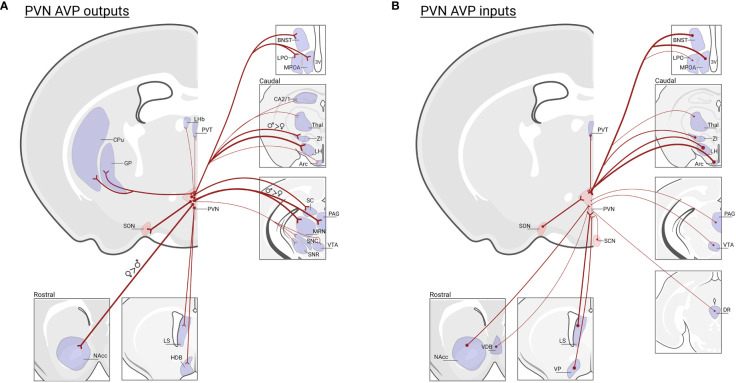
*PVN AVP cell inputs and outputs in mice*. **(A)** Regions that receive prominent input from vasopressin cells in the PVN: (NAcc)* nucleus accumbens; (LS) lateral septum; (HBD) horizontal diagonal band; (MPOA) medial preoptic area; (LPO) lateral preoptic area; (BNST) bed nucleus of the stria terminalis; (LHb) lateral habenula; (PVT) paraventricular nucleus of the thalamus; (GP) globus pallidus, (CPu) caudate putamen; (SON) supra optic nucleus; (CA2/1) hippocampus;(Thal) thalamus; (ZI)† zona interna; (LH) lateral hypothalamus; (Arc) arcuate nucleus; (SC) superior colliculus; (PAG)†, periaqueductal gray; (MRN)†, median raphe nucleus; (SNC) substantia nigra pars compacta; (SNR) substantia nigra reticulata; (VTA), ventral tegmental area; Data based on PVN AVP cell-specific tracing (125) and the location of AVP-ir fibers remaining after male gonadectomy or SCN lesions (46). Note that fiber measurements do not determine AVP cell synapses in labeled regions. **(B)** Regions that send input to vasopressin cells in the PVN: NAcc, vertical diagonal band (VDB), LS, ventral pallidum (VP), BNST, MPOA, LPO, PVT, SON, suprachiasmatic nucleus (SCN), Thal, ZI, LH, Arc, PAG, VTA, dorsal raphe (DR); data based on transsynaptic-monosynaptic retrograde tracing from PVN AVP cells (127). Not all PVN AVP cell input/output regions are listed. Thickness of lines represents the strength of projections. * Indicates greater projections in females, † indicates greater projections in males. Created with BioRender.com.

AVP is produced in magnocellular PVN and SON neurons that project to the posterior pituitary where they release AVP into the periphery to regulate, e.g., blood pressure and water balance (121, 122). AVP is also expressed in parvocellular PVN neurons that project to the median eminence, where AVP is released into a portal system between the median eminence and the anterior pituitary to regulate adrenocorticotropic hormone (ACTH) from the corticotrophs, and thereby indirectly release of glucocorticoids from the adrenal cortex (123). A separate group of parvocellular neurons project centrally to brain regions to modulate physiology as well as social and other motivated behaviors (8, 12, 124). PVN AVP neurons, while not differing in cell number in males and females (46), do display projection patterns that partially differ by sex (125). In females, PVN AVP cells send stronger projections to striatal areas (i.e., nucleus accumbens; NAcc) whereas males have denser innervation of mid- and hind-brain regions (125). Moreover, PVN AVP cell projections are generally denser in females compared to males (46). As many of these regions contain both V1aR and OXTR (126), AVP released from PVN cells may act on both V1aR and OXTR-expressing neurons to drive behavior, given the known cross-talk between OXT/AVP receptors (29). Not only do PVN AVP cells project to a variety of regions, they also receive input from brain regions that regulate aspects of social and emotional behavior, such as the ventral tegmental area (VTA), NAcc, dorsal raphe (DR), BNST, LS, VP, and preoptic area (POA) (127) (**Figure 3**). Even though PVN and BNST/MeA AVP cell projections sometimes overlap, regions that regulate prediction of aversive and reward outcomes (i.e., lateral habenula and lateral septum) receive much stronger AVP input from the BNST/MeA compared to the PVN (46, 128) (**Figure 1B**).

## Function of PVN AVP cells

### Opposite-sex interactions (affiliation)

Forming pair bonds increases AVP-ir in the PVN of male and female zebra finches (70), whereas pair-bond breakage with concomitant increases in anxiety-like and affiliative behavior also increases PVN AVP-ir in prairie voles (129). In birds and prairie voles, parenting and nesting behavior are consistently positively correlated with levels of PVN AVP expression and cellular activity (130–132).

### Same-sex interactions (aggression)

The relationship between PVN AVP dynamics and aggression are inconsistent. For example, dominant male, but not female, Mandarin voles have greater PVN-AVP-ir than subordinates (133), and aggressive behavior is positively correlated with this increased AVP expression in Brandt’s voles (134). In male song sparrows, PVN AVP cells are more active after agonistic encounters and, in lizards, PVN AVP cell activity is positively correlated with aggression and can predict subordinate status (73, 135). However, PVN AVP-ir levels do not differ between males of aggressive and less aggressive Peromyscus species (86), and, in male mice, PVN AVP-ir is reduced following aggressive interactions (136). It is clear that responses of PVN AVP cells in agonistic contexts are complex, varying with social context, sex, personality, and their interactions (137).

### Social stress and aggression

A number of studies have focused on AVP interactions with the hypothalamic–pituitary–adrenal (HPA) axis (138, 139). The HPA axis is activated during aversive situations to facilitate both physiological and behavioral coping responses/strategies (140). Following HPA activation, animals may display increased vigilance and arousal, and chronic HPA activation can lead to psychiatric disorders (i.e., anxiety and depression) in humans (141, 142). During stress, hypothalamic and systemic release of AVP are regulated in a site- and stressor-specific manner. For example, parvocellular PVN AVP cells can regulate ACTH secretion, which ultimately increases corticosteroid release (138). In addition to its role in the regulation of general stress physiology, several lines of evidence link PVN AVP cells to behavioral responses to social stress and the regulation of anxiety-related behaviors. AVP is released within the PVN of rats following social defeat, without stimulating the hypothalamic–neurohypophysial system or HPA axis (43, 140), implicating local somatodendritic AVP release. In resident-intruder tests, intruder rats that used active, but not passive (e.g., freezing behavior) coping strategies, increased AVP release in the PVN (140). In contrast, the effects of chronic social stress on AVP expression within PVN are mixed, if not contradictory. For example, following long-term social defeat, both increases and reductions in PVN *Avp* mRNA (or no change in PVN AVP-ir) are observed in male mice (143, 144). Moreover, changes in PVN AVP expression following chronic social defeat have not been detected in male rats (145), and even acute social defeat has little effect on PVN *Avp* gene expression in mice (143, 144). However, acute social defeat does reduce PVN *Avp* mRNA and AVP immunoreactivity in male, but not female, California mice (40). These changes in California mice are not correlated with changes in aggression but are correlated with a reduction in social investigation. Overall, it is unclear if species differences or differences in short versus long-term AVP action are responsible for these conflicting results about the role of PVN AVP cells in social stress.

The species-specific nature of PVN AVP signaling following social stress are paralleled by findings that PVN AVP responses following non-social stress also depend heavily on the species investigated. Rats and mice bred for high anxiety traits (i.e., passive coping) had greater Avp gene and AVP peptide expression within the PVN compared to low-anxiety lines (146–151); an effect which is found in male, but not female rats (149, 150). Additionally, *Avp* mRNA increases in the PVN after acute stress in rats (43, 152). However, this type of increase is not observed in prairie voles following swim stress (153), and, in fact, reductions in *Avp* mRNA occur in female mice following acute restraint (154), indicating that species, sex, and type of acute non-social stressor determine the level of *Avp* gene expression in PVN. Similar species differences are also apparent in PVN AVP responses to chronic stress. Female mice, but not male mice, show decreased *Avp* mRNA (155), whereas California mice show increases in *Avp* mRNA after chronic variable stress (156). Notably, neither acute nor chronic restraint in male rats or chronic variable stress in mice alters AVP immunoreactivity (155, 157). This may be due to significant heterogeneity in PVN AVP cell responses such that different AVP cell groups within the PVN may respond in opposite directions to chronic stressors (158).

The lack of agreement about PVN AVP stress-related signaling across studies might reflect not only known sex- and species-differences but also unresolved mechanisms. For example, because PVN AVP cells can release AVP from soma and dendrites independently from synaptic release (51), AVP levels within the PVN may reflect non-synaptic, possibly autoregulatory, local release, rather than release of AVP in PVN target structures. Indeed, high anxiety rat lines do not increase AVP release in the PVN during maternal aggression but do increase it in the central amygdala (CeA), where V1aR antagonists can reduce maternal aggression (159). This may explain the conflicting effects of AVP agonists/antagonists injected into PVN on anxiety-like behavior (43, 149, 160). Additionally, changes in PVN AVP cell activity and AVP production may reflect physiological adaptations linked to behavioral changes but may not be causally involved in driving these behaviors. Lastly, because the PVN contains several different AVP cell types (i.e., magnocellular and parvocellular), it is likely that these different cell populations could have different effects on behavioral responses to social and non-social stressors and may themselves be composed of heterogeneous subpopulations, such as the PVN AVP cell population that expresses estrogen-receptor beta primarily in females (161, 162). Consequently, intersectional genetic approaches, such as those used to study PVN OXT (126, 163), may be useful in identifying the function of specific PVN AVP cell subpopulations in social stress.

### Developmental aspects of social behavior (social play) and parental behavior

In addition to their responses following social stress and aggression, PVN AVP neurons also respond in other social contexts. Avp mRNA levels in the PVN are positively correlated with social play in male, but not female, juvenile rats (97, 164). Likewise, increases in PVN Avp mRNA are positively correlated with levels of adult male-male interactions in prairie voles and mice (165, 166) and with parental care (both sexes) in prairie, but not montane, voles (130). Taken together, PVN AVP levels/activity appears to be correlated with changes in both agonistic and affiliative interactions, with the direction of the correlation largely depending on the species examined.

## Direct manipulation of PVN AVP cells

### Opposite- and same-sex interactions (aggression and social investigation)

Although there have been a number of correlational studies suggesting PVN AVP’s involvement in aggression (see above), direct manipulations of PVN AVP cells suggest that these cells are not required for aggressive behavior. In mice, ablations of AVP-expressing cells in the PVN did not alter resident-intruder aggression (119), and PVN AVP knockdown in zebra finches did not alter same-sex aggression (137). Therefore, other hypothalamic AVP-expressing cell groups, such as the nucleus circularis (NC) (see below), may be driving pro-aggressive effects of AVP (167–169).

PVN AVP cells may have a greater influence on female social interactions compared to males. For example, PVN AVP cell ablations increase female, but not male, social approach and investigation in mice (119). Similarly, chronic variable stress increases social investigation in female, but not male, mice while concomitantly reducing Avp mRNA levels in PVN (155). In addition, PVN AVP action on CRF cells can mediate the effects of social buffering in female mice, effectively “erasing” the synaptic effects of a stressful experience (170). Therefore, AVP within the PVN may regulate, among other things, female stress resilience.

### Social memory

Unlike the effects of BNST AVP cell deletions, removing PVN AVP cells did not affect the ability of mice to distinguish between familiar and unfamiliar mice (171). However, activation of PVN AVP afferents to the CA2 subfield of the hippocampus enhances social memory in male mice (172). This discrepancy between results may be due to procedural differences in behavioral testing but may also indicate that, while this circuit is sufficient to modulate memory, it may not be absolutely required. Alternatively, critical PVN AVP sub-circuits, such as those projecting to CA2, may have been spared in the lesion experiment.

### Parental behavior

Recent work in monogamous mice has demonstrated that chemogenetic inhibition of PVN AVP cells increases nest building in male mice, while excitation reduces nest building in females, without altering any other aspects of parental behavior (173). These sex differences in PVN AVP function may be examples of compensation for other biological sex differences as monogamous male and female mice display similarly high levels of nest building and parental care (174).

### Anxiety and social interactions

In addition to social behavior, PVN AVP cells contribute to sexually differentiated aspects of anxiety-like behavior. For example, removal of these cells increases non-social, anxiety-related behaviors in males, but not in females (119). In contrast, administration or knockdown of AVP in the PVN does not alter anxiety-like behavior in male rats or male and female zebra finches (137, 160). The lesion results also diverge from some correlational studies showing that PVN AVP-ir positively correlates with levels of anxiety-like behavior in rats and mice (148, 149, 151), and that reductions of PVN AVP expression correlated with increases in anxiety in juvenile male, but not female, rats (175). Collectively, however, these data suggest that AVP within the PVN may influence anxiety-like states primarily in males, while not affecting their ability to socially interact with male and female conspecifics, suggesting a lack of linkage between social- and non-social anxiety states (119).

## Vasopressin cells of the SON and nucleus circularis

Although the peripheral effects of SON AVP on physiology are well known (176), much less is known about the behavioral function of these cells. Changes in measures of cellular activity in SON AVP cells suggest that they may modulate aggressive behavior or aggression-related physiology in rats and hamsters (169, 177, 178). More directly, non-selective lesions of the medial SON impaired odor-induced flank marking in hamsters (179), suggesting that a subpopulation of SON AVP cells may drive aspects of social communication.

The nucleus circularis (NC) is a small cluster of cells that contain AVP located within the anterior hypothalamus between the SON and PVN and has been historically implicated in osmotic thirst (180, 181). The discovery that AVP release within the anterior hypothalamus (AH) drives social communication (i.e., flank marking) in male hamsters (182) and that non-selective NC lesions reduces flank marking, led to the suggestion that these cells could be one of the AVP sources important for flank marking (179). Indeed, in male hamsters, the number of NC AVP-ir neurons positively correlates with offensive aggression, social dominance status, and flank marking (167, 177, 183), and, in prairie voles, the number of cells correlates with the level of mating-induced aggression (50, 168, 184). This may explain why PVN and BNST AVP cell lesions in mice did not affect aggressive behavior in resident-intruder settings (106, 119). However, as lesions to NC were non-specific, damaging both NC and the nearby AH, i.e., the location where AVP drives flank marking in males (182, 185), it is difficult to discern whether AVP production within the NC drives flank marking. Specific manipulations of NC AVP cells, such as those used to assess BNST AVP function (106), will be necessary for determining their role in social behavior.

## Vasopressin cells of the SCN and retina

AVP is also produced within the suprachiasmatic nucleus (SCN), a key driver of circadian rhythms in behavioral and physiological regulation (186–188). The AVP cells, located in the dorsomedial SCN, receive their inhibitory inputs from the ventrolateral SCN, which, in turn, is targeted by a subpopulation of retinal ganglion cells some of which also express AVP (189). SCN AVP release, as well as the expression of genes involved in photo-entrainment of biological rhythms, likely mediate aspects of jetlag (189, 190). SCN AVP neurons project locally as well as to the PVN, the subparaventricular zone, MPOA, BNST, PVT, ARC, dorsal medial hypothalamus, NTS, and into the cerebrospinal fluid (CSF) (46, 186, 188, 191).

Although it has been suggested that SCN AVP may interact with sex hormones to influence sexual preference in humans (192), the direct influence of SCN AVP on social behavior is much less clear. Non-specific SCN lesions in male Syrian hamsters did not influence AVP-sensitive social communication (i.e., flank marking) (193). Lesions of AVP cells in SCN of mice did not influence their social behavior but did increase anxiety-like behavior and sucrose consumption in both sexes (194). However, since these lesions only reduced the number of SCN AVP cells by about 50%, greater reductions in the population of SCN AVP cells may reveal additional effects on social behavior.

## Vasopressin cells in the olfactory system

In rats, the main (MOB) and accessory (AOB) olfactory bulbs contain projection neurons that express AVP (195, 196). These AVP cells are non-bursting, tufted cells (31), which extend their primary dendrites toward glomeruli, thereby receiving information from olfactory sensory neurons (195). Some of these AVP cells also co-express V1bR and may therefore be strongly autoregulated (196). In the AOB, AVP cells project to MePD, which may influence kisspeptin release within this region (which also contains AVP cells) (197). In addition, AVP neurons have also been found in the olfactory cortex (anterior olfactory nucleus, piriform cortex), indicating their involvement in complex odor recognition (31, 195).

In most mammals, olfactory processing is critical for appropriate social communication *via* chemosignals produced by a conspecific (198). Pharmacological work suggests that AVP is released in the OB to modulate early stages of sensory processing and social recognition (195) *via* feedback inhibition, a key component in sensory processing (199). As with other OB projection neurons, AVP cells are modulated by cholinergic systems to ultimately regulate social odor processing (200). More work is needed to determine the specific excitation, inhibition, or disinhibition pathways within which OB AVP cells function to modulate social interactions.

## AVP action in humans and other primates

Like humans, many non-human primates are highly social and capable of complex social cognition, making them desirable models for studies of social competence. As in other species, AVP and V1aR is expressed in social brain regions of primates (201–204) and is likely to influence their social behavior. For example, AVP concentration in CSF, but not CSF OXT or blood AVP concentration, predicts social functioning in Rhesus monkeys and positively correlates with time spent in social grooming (21, 205). In humans, manipulation of AVP and V1aR systems have been explored as therapies for ASD, schizophrenia, and drug-abuse (21, 53, 206, 207). Indeed, CSF AVP is linked to ASD symptom severity in children (208) and blood AVP concentration may be useful in predicting social development outcomes in newborns (21). Intranasal AVP also increases risky cooperative behavior in men (209) and enhanced social skills in autistic children (210). Additionally, intranasal AVP has been shown to modulate emotional responses to faces (211, 212), improve memory for emotional faces, and identification of social words (5, 21, 213). Although intranasal administration of AVP may offer a promising future therapeutic, the mechanisms by which AVP acts to modulate social behavior in humans remains to be seen. This will be especially important because of sex differences in ASD diagnoses (21) and sex-specific effects observed in animal models. In none of these cases, it is clear which source of AVP contributes to AVP effects on social behavior.

## Conclusions

**Figure 4:**
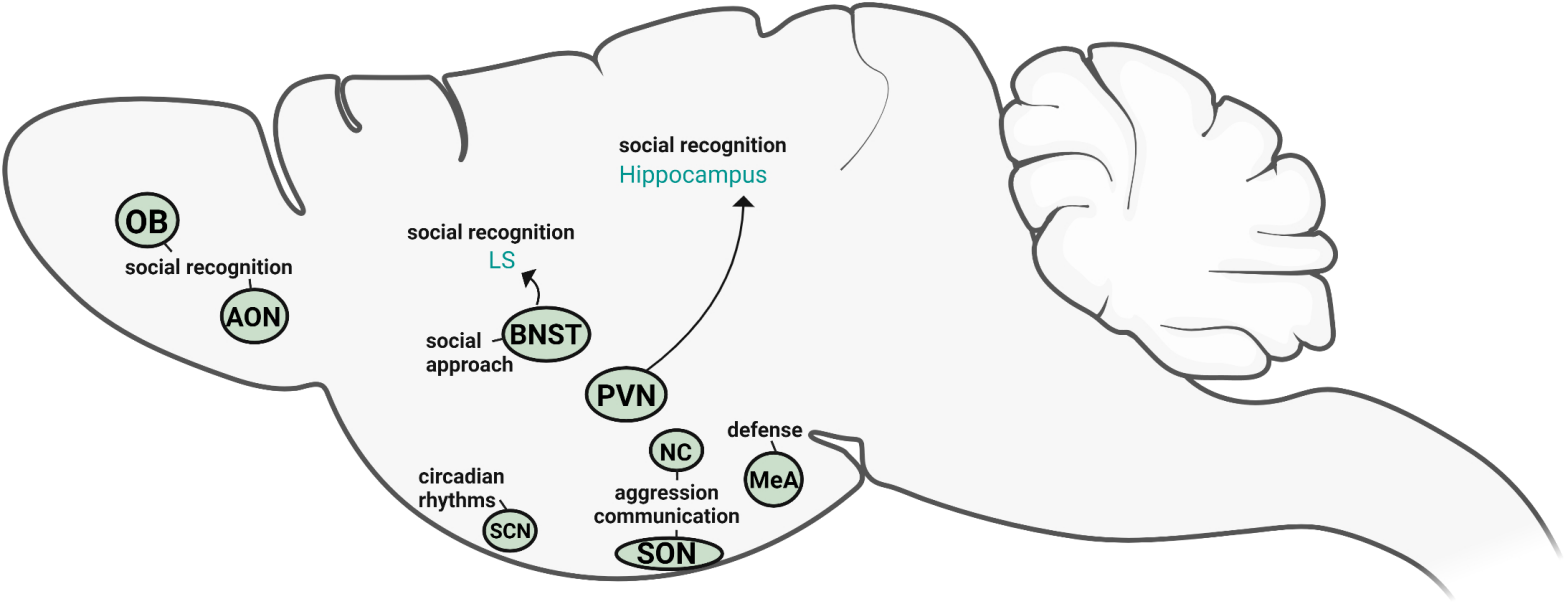
*Sources of vasopressin relevant for social behavior*. Based on the available direct evidence, the AVP cells within PVN, BNST, OB, and AON directly influence social recognition and social approach, whereas NC and SON AVP cells modulate aggression and competitive signaling. MeA AVP cells primarily regulate defensive responses. SCN AVP cells regulate circadian rhythms but are not directly linked to social behavior. Created with BioRender.com.

AVP in the nervous system originates from several distinct sources which are, in turn, regulated by different inputs and regulatory factors. Based on both direct and indirect evidence, we can begin to define the specific roles of AVP cell populations in social behavior (**Figure 4**). The cell population that has been most directly linked to social behavior is the sexually differentiated, steroid-sensitive BNST AVP cells. This population appears to be a likely driver of male-typical social investigation (of potential competitors) and social recognition, as well as regulators of affiliative responses (pair bonding, flocking, sexual behavior). Although much less is known about the other sex-different, steroid-sensitive AVP population in the MeA, it too may be involved in social investigation as well as defensive behavior. The BNST and MeA AVP cells send projections to several regions known to regulate social behavior (32); however, it is unknown if these projections originate from the same or different AVP cell populations within the BNST/MeA. Paradoxically, one of the largest AVP cell populations, the PVN, has the least amount of direct evidence for its role in social behavior. The indirect evidence that is available presents a rather inconsistent picture of the role of PVN AVP cells in social behavior and may, at best, suggest a modest role for PVN AVP cells in the behavioral responses to stress. In contrast, direct manipulations of PVN AVP cells in mice suggest that these cells normally suppress inappropriate social/emotional behavior: social investigation in females and anxiety-like behavior in males. However, the specific role that PVN AVP cells play in social behavior may depend heavily on species- and social system- differences. For example, PVN AVP may promote social affiliation in gregarious bird species of both sexes as well as suppressing inappropriate (mate-directed) male aggression. While the available evidence suggests a minor role for PVN, BNST, and MeA AVP cells in aggressive behavior, the reputation of AVP as a pro-aggressive neuropeptide in males rests almost exclusively on AVP action within the anterior hypothalamus, with the NC and parts of the SON likely contributors of behaviorally-relevant AVP. More recently, AVP expression within early segments of the olfactory systems has been shown to facilitate odor-based social recognition. Consequently, AVP may facilitate social odor memory by both biasing social odor processing (olfactory bulb and cortex) and by modulating memory consolidation through more central circuits (BNST-LS, PVN-hippocampus). Even though AVP in the SCN plays an important role in adjusting circadian rhythms, it does not appear to play a critical role in social behavior but may, instead, regulate reward and anxiety-like behavior. How (or whether) these different AVP systems interact in influencing social behavior is completely unknown.

**Figure 5:**
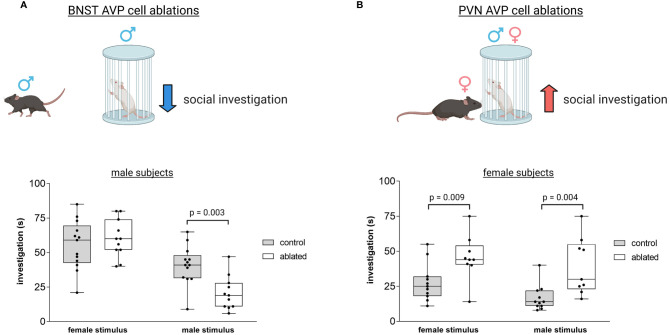
*Distinct vasopressin cells modulate social investigation in a sex-specific manner*. **(A)** BNST AVP cell ablations in males reduce male-male social investigation [redrawn from (106)]. Boxplot and individual data points of time spent investigating male or female stimulus versus clean empty cage within the three-chamber apparatus. p = 0.003. A significant interaction was found between genotype and sex (F(1,41) = 4.9, p = 0.03. **(B)** PVN AVP cell ablations increase female social investigation of male (p = 0.004) and female (p = 0.009) stimuli compared to controls to similar levels as male social investigation [redrawn from (119)]. A significant interaction was found between genotype and sex (F(1,44) = 5.33, p = 0.02). Created with BioRender.com.

Several themes and conclusions emerge from examining the evidence implicating different AVP sources in social and emotional behavior. First, because different AVP systems project to overlapping or contiguous brain regions, limiting analysis to sites of AVP action prevents a complete understanding of how AVP regulates social behavior (42, 214). Similarly, as indirect measures of AVP action often show contradictory results (particularly in the PVN), direct tests of AVP cell population function are needed to clarify how different systems function across the neural axis. Second, the few studies that have tested the function of specific AVP cell projection zones have only examined a limited set of social behaviors (social investigation, communication, and recognition). Consequently, the role of AVP sources in social processing must be expanded to include other aspects of social behavior, different contexts, and other model organisms, preferably using comparable and robust measures of function. Lastly, it is clear that sex differences in function may be apparent in both sexually-dimorphic structures (BNST/MeA) as well as ones without prominent structural differences (PVN). For example, PVN and BNST AVP cells in mice control social and emotional behavior differently in the two sexes (106, 107, 119) (**Figure 5**), such that BNST AVP cell ablations decrease male social investigation of other males (106) (**Figure 5A**), whereas PVN AVP cell ablations increase only female social investigation of males and females (119) (**Figure 5B**). This suggests that the PVN may contribute to baseline sex differences in social investigation, since males show higher levels of investigation than do females (90, 215–217) and removal of these cells removes the sex difference in social investigation (119).

The application of modern molecular approaches will greatly increase our basic understanding of how and where AVP acts to regulate social behavior, much in the same way as it has transformed research on oxytocin systems (126). This increasingly precise understanding of how AVP systems are organized and function may ultimately lead to better therapeutic interventions for psychiatric disorders characterized by substantial social deficits.

## Author contributions

NR wrote the review article, GD and AP provided feedback and edited the article. All authors contributed to the article and approved the submitted version.
